# Clinico-epidemiological profile and redox imbalance of lung cancer patients in Algeria

**DOI:** 10.25122/jml-2018-0041

**Published:** 2018

**Authors:** Amina Otsmane, Ghouti Kacimi, Saida Adane, Farid Cherbal, Souhila Aouichat Bouguerra

**Affiliations:** 1. Molecular and Cellular Physiopathology Team, Biology and Physiology of Organisms; 2. Mohamed Nekkache Hospital, 16205; 3. Department of Medical Oncology, Mohamed Nekkache Hospital, 16205; 4. Unit of Genetic, Laboratory of Molecular and Cellular Biology, Faculty of Biological Sciences, University of Sciences and technology Houari Boumediene, 16011

**Keywords:** Lung cancer, clinico-epidemiological profile, redox imbalance, Algeria, AOPP – Advanced Oxidation Protein Products, LC – lung cancer, MDA: – Malondialdehyde, NO – Nitrogen monoxide, NSCLC – Non-Small Cell Lung Cancer, OS – oxidative stress, ROS – Reactive Oxygen Species, SCLC – Small Cell Lung Cancer

## Abstract

**Hypothesis:** How are the epidemiologic repartition and the physiopathology of lung cancer (LC) in Algeria?

**Objective:** Our study aimed to establish the clinico-epidemiological profile and evaluate redox imbalance in Algerian patients with LC.

**Methods and results:** Our study concerned 94 Algerian patients with LC treated at two hospitals of Algiers, the capital of Algeria. The clinico-epidemiological profile was established. Moreover, the redox imbalance was evaluated by dosing oxidative stress (OS) parameters in tumor tissues and blood. We noted that the average age was 62.06 years, and 79 among the 94 patients were male, 94.59% of which were smokers. The most common histological type was adenocarcinoma (45.45% of cases), followed by squamous cell carcinoma (37.88%) small-cell carcinoma (4.86%) and other histological types (6.67%), while the most frequent clinical stage was IV (66.95 %). 23 of the 94 patients were exposed to particular risk factors such as masonry products, metal mechanics, coal smoke and so forth. In other respects, the OS parameters: NO (Nitrogen monoxide), AOPP (Advanced Oxidation Protein Products) and MDA (Malondialdehyde) were higher in tumor tissues compared to peritumoral stroma (control), unlike the catalase activity. Otherwise, AOPP and MDA were significantly higher in patients’ blood than in healthy control blood, in contrast to the catalase activity.

**Discussion:** The LC has a heterogeneous repartition regarding the sex, age, histological types, the smoking status and professional exposition to risk factors in the Algerian population. Moreover, the oxidative stress impacts the physiopathology of LC.

## Introduction

Lung cancer (LC) is the most widespread cancer in the world; according to the International Agency for Research on Cancer, 1.8 million new cases were diagnosed in 2012, 58% of which occurred in the less developed regions. According to the national cancer plan established by Professor Zitouni in 2014, LC is a major health problem and the most common form of cancer in Algeria. Also, it represents 15% of male cancers.

The lung is an interface of exchange with the environment hence it is the most targeted organ by higher exogenous oxidants such as cigarette smoke components [1]. Active or passive exposure to cigarette smoke constitutes the major risk factor of LC [2]. Endogenous oxidants are physiologically produced in small quantity by organisms and play numerous important vital roles [3]. However, in pathological conditions, oxidants amounts increase and induce a redox imbalance generating an oxidative stress (OS) which induces several biomolecular modifications [4] such as lipid peroxidation [5] and protein attacks (oxidation, glycation or conjugation) [1], the OS being implicated in the pathogenesis of several pathologies, especially in cancer [6,7].

## Patients and Methods

### Recruitment

In this study, 94 LC patients were recruited in services of thoracic surgery at Mohamed Nekkache Hospital and University Hospital Mustapha Bacha, Algiers, Algeria. It included patients from 27 different provinces and they did not receive any anticancerous therapy. Algeria is a large country in North Africa, is the largest country of the Mediterranean Sea, the largest on the African continent and the tenth-largest country in the world.

### Ethical disclosure

This human study was performed in accordance with the Declaration of Helsinki and it was also approved by the institutional/national committee. All adult participants provided written informed consent to participate in this study.

### Data collection and samples/control preparation

Epidemiological and clinical data included age, gender, socioeconomic status, smoking status and histological types and were obtained to establish the epidemiological profile. Furthermore, the OS parameters were dosed in tumor and serum samples. We collected 63 blood samples from LC patients; the blood samples were collected on a dry tube, centrifuged at 3000 g for 10 minutes and sera were collected. Moreover, 46 fresh tumor specimens were obtained from 46 NSCLC patients who had undergone surgical resection. Those specimens were frozen directly in liquid nitrogen, crushed, divided into two aliquots and immersed separately in two buffer types (standard extraction buffer and MDA buffer), then centrifuged at 3000 g for 10 minutes. The supernatants (tissue extracts) were collected. Both sera and tissue extracts were conserved at 20C° up to the time of analysis.

Serum values of the patients were compared to control values which were collected from 12 healthy individuals. Tumor extract values were compared to the value of the peritumoral stroma as a tissue control which was obtained from 10 LC patients. The collection and treatment of control tissues and blood specimens were similar to those of the samples.

### Clinico-epidemiological profile

The collected clinico-epidemiological data were distributed according to the most common risk factor: cigarette smoke.

### OS parameters levels measure

#### Nitrogen monoxide: NO

NO was evaluated according to the Griess method, based on the evaluation of nitrates and nitrites which form a diazonium salt with sulfamic acid that couples with N-naphthylethylene diamine [8]. NO is expressed in μmole/g of tissue and is evaluated from Sodium nitrite standard curve.

#### Malondialdehyde: MDA

MDA was quantified colorimetrically using Thiobarbituric Acid (TBA): one molecule of MDA binds with two molecules of TBA to form Thiobarbituric Acid Reactive Substances (TBARs) [9]. MDA is expressed in μmole/g of tissue and obtained from the standard MDA curve.

#### Advanced Oxidation Protein Products: AOPP

AOPP were quantified using the iodometric method based on the oxidation of Potassium Iodide in an acidic environment [10]. AOPP is expressed in μmole/g of tissue and determined from the chloramine-T standard curve.

#### Catalase

Catalase activity was measured according to Aebi method’s (1984) by the measurement of H_2_O_2_ disappearance speed [11]. Catalase is expressed in UI/g of tissue and defined by the following formula: Catalase (UI/g) = [2,3033/(T1-T0) x log (ODo/ODT)] /g of total proteins

* Catalase and AOPP rates are reported to total proteins rates’ which were evaluated by Bradford assay.

#### Statistics

Results are expressed as the mean ± standard deviation. All tests were performed using the XLSTAT software (version 2014.5.03). Comparison of OS parameters levels between samples and control were examined using the Mann-Whitney test.

P-value <0.05 was considered as statistically significant.

## Results

### Clinico-epidemiological data

We noted that in our study, LC has a heterogeneous repartition; the average age of implicated patients was 62.06 years old, and 79 among the 94 patients were male, the sex ratio being higher than 4.87.

**Table 1:** Clinicopathological and epidemiological characteristics of recruited Algerian patients with lung cancer

Characteristics		Exposure		
		Non-smokers (%) n= 20	Smokers (%) n= 74	Group (%) n= 94
**Gender**	Male	15.26	94.59	88.58
	Female	84.74	4.05	16.58
**Age (years)**	Age range	[16 – 81]	[36 – 81]	[16 – 87]
	Middle age	53.651 ± 19.91	64.34 ± 10.13	62.06 ± 12.85
**Diagnosis**	NSCLC	95	93.24	95.14
	SCLC	5	6.76	4.86
**Histological type of NSCLC**	Adenocarcinoma	70	50	45.45
	Squamous cell carcinoma	20	41.94	37.88
	Others types	10	8.06	6.67
*Stages*	*Stage I*	—	1.75	1.56
		14.28	—	1.56
	*Stage II*	14.28	8.77	9.37
		14.28	12.28	12.5
	*Stage III*	28.57	12.28	14.06
		-	15.79	14.06
	*Stage IV*	40.81	49.12	66.95
**Signs and Symptoms**	Persistent cough	42.86	61.90	47.73
	Chest pain	73.06	41.27	43.54
	Weight-loss	14.29	25.40	19.32
	Dyspnea	-	22.22	14.77
	Hemoptysis	28.57	25.40	19.32
	Fever	-	7.94	5.68
	Anorexia	14.29	-	1.14
	Dysphagia	-	1.59	1.14
	Tiredness	14.29	7.94	6.82
	Paraneoplastic syndrome	-	3,17	3.41
	Metastasis pain	20.41	4.76	8.28
**Family medical story**		14.29	4.05	5.68
**Exposition to domestic and professional risk factors**	Masonry/construction products	-	13.51	11,36
	Mechanics of sheet metal	-	8.11	6.82
	Mines	-	4.05	3,41
	Coal smoke	14.29	2.70	4,54
**Cigarette consumption**	Active smoking	-	94,20	94,20
	Passive smoking	-	5.80	5.80
**Frequency of cigarette consumption (cigarettes/day)**	High (>20)	-	54.17	54.17
	Moderate ([5-20])	-	48.83	48.83
	Low (<5)	-	-	-
**Quitting smoking**	Stop smoking	-	59,42	59,42
	Don’t stop smoking	-	40,58	40,58
**Respiratory diseases**	COPD	-	2.70	2.27
	Tuberculosis	-	2.70	2.27
	Pleurisy	-	1.35	1.14
	Pneumothorax	-	1.35	1.14
	Pleural effusion	-	1.35	1.14
	Asthma	14.29	2.70	4.54
	Allergic rhinitis	-	1.35	1.14
**Metabolic diseases and complications**	Diabetes	28.57	18.92	20.45
	HBP	21.43	24.32	23.86
	Cardiopathy	-	9.46	7.95
	Dyslipidemia	-	3.40	4.54
**Endocrine diseases**	Prostate adenoma	-	8.11	6.82
	Dysthyroid	7.14	1.35	3.41

**COPD:** Chronic Obstructive Pulmonary Disease; **HBP:** High Blood Pressure; **NSCLC:** Non-Small Cell Lung Cancer; **SCLC:** Small Cell Lung Cancer.

**Table 2:** Oxidative stress parameters in tissues and serum in Algerian patients with LC. Comparison between tumor tissue extracts and peritumoral stroma and between patients’ blood and healthy control’s blood

**Parameter**		**Tissues**			**Blood**	
	*Tumor tissue extracts*	*Control extracts*	*p Mann-Whitney*	*Lung cancer patients*	*Healthy control subjects*	*p Mann-Whitney*
**AOPP (μmole**)	36.4115.87±	1.17±0.38	<0.0001 (****)	48.22±17.26	15.09±4.51	<0.0001 (****)
**MDA (μmole)**	5.12±3.65	3.96±1.48	0.797 *(p>0.05)*	1.12±0.64	0.24±0.15	<0.0001 (****)
**NO (μmole)**	90.06±25.69	60.87±21.68	<0.0001 (****)	—	—	—
**Catalase (UI)**	2.87±1.35	5.37±2.82	0.008 (**p<0.01)	12.35±10.38	51.43±34.61	0.002 (**p<0.01)

84% of patients were smokers, of which 94.59% were male. NSCLC was the most common diagnosis (95.14%) in both non-smokers and smokers patients, where adenocarcinoma was the most represented histological type (45.45%) followed by squamous cell carcinoma (37.88%) and small-cell carcinoma (4.86%). The most frequent clinical stage was IV in both smoking (46.87%) and non-smoking patient groups (40.81%). We noted that cigarette smoke is considered the most important factor (84.09%), but 23 among the 94 patients were also exposed to particular risk factors such as masonry products, mechanic metals and coal smoke. Genetic predisposition was also noted to be essential in non-smokers.

Also, many signs and symptoms were reported, particularly persistent cough, chest pain, weight loss, dyspnea and hemoptysis. Regarding the medical history and/or associated diseases, respiratory and metabolic diseases were the most noted in both smokers and non-smokers LC patient’s (Table 1).

### OS parameters

**Figure 1:**
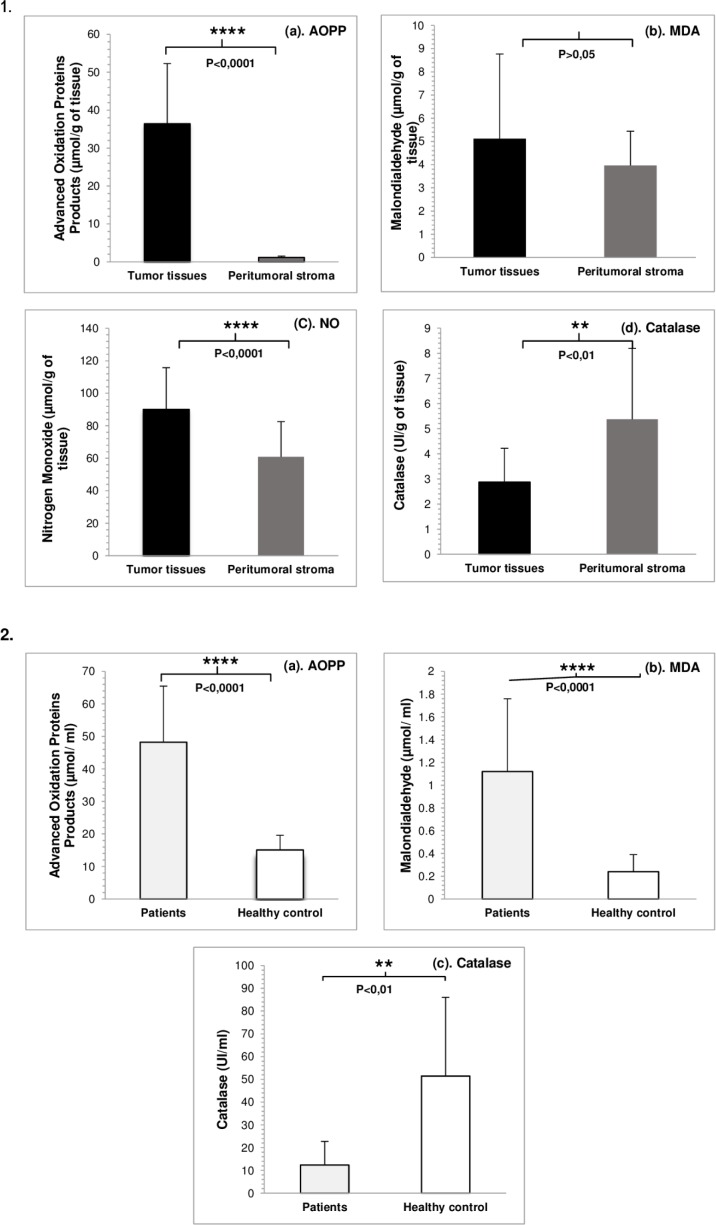
Values of oxidative stress parameters and statistical significance in samples compared to control in 1. Tissue extracts (a. AOPP; b. MDA; c. NO; d. catalase) and 2. Blood (a. AOPP; b. MDA; c. catalase), in LC patients. 1. Tissue extracts: (a)****p<0.0001: AOPP tumor tissues vs AOPP peritumoral stroma, (b). p>0.05: MDA tumor tissues vs. MDA peritumoral stroma; (c). ****P<0,0001: NO tumor tissues vs. NO peritumoral stroma; (d).**p<0,01: catalase tumor tissues vs. catalase peritumoral stroma. 2. Blood: (a). ****p<0.0001: AOPP patients vs AOPP healthy control; (b). ****p<0.0001: MDA patients vs. MDA healthy control (c). *** p= 0.002: catalase patients vs. catalase healthy control.

The assessment of tumor tissue extracts and serum OS markers showed a redox imbalance characterized by an increased level of oxidant/oxidation products and a decreased level of antioxidants in both tumor tissue extracts and patients’ blood compared to peritumoral stroma (control) and healthy control blood, consecutively (Table 2; Figure 1).

Tissue evaluation of OS revealed that compared to control, tumor extracts had a significantly increased level of AOPP (p<0.0001) and NO (p<0,0001) but a non-significant MDA level (p>0,05). Catalase level was non-significant decreased in tumor extracts compared to control group (p<0,01) (Figure 1.1).

Furthermore, in the OS serum evaluation, we showed a significantly increased level of both MDA (p<0.0001) and AOPP (p<0.0001) and a significantly decreased level of catalase (p=0.002) compared to healthy control group (Figure 1.2).

**Table 3:** Does cigarette smoking influence the redox imbalance? Comparison of oxidative stress parameters between tumor tissues of Algerian smokers patients with LC and control tissue.

Parameter	Tumor tissue *extracts*	Control *extracts*	p *Mann-Whitney*
**AOPP (μmole)**	35.15±15.87	1.17±0.38	*<0.0001* (****)
**MDA (μmole)**	4.17±3.21	3.96±1.48	0.454 (p>0.05)
**NO (μmole)**	91.65±23.77	60.87±21.68	<0.0001 (****)
Catalase (UI)	2.88±1.56	5.37±2.82	0.015 (*p<0.05)

**Figure 2:**
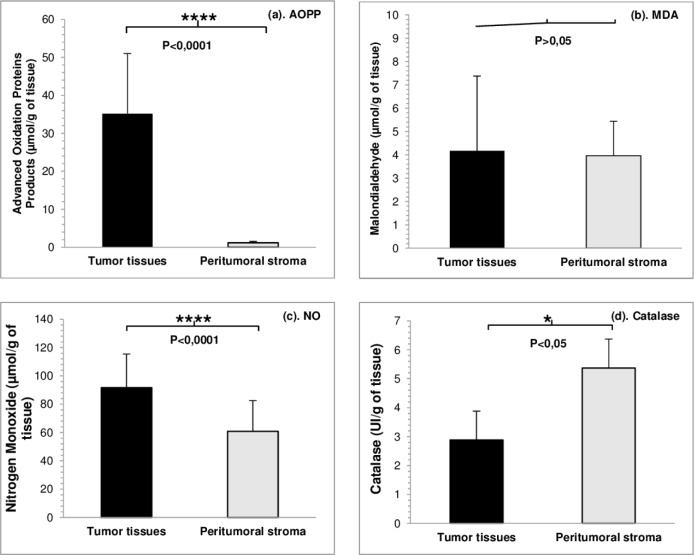
Values of oxidative stress parameters and statistical significance in smokers’ tumor tissue compared to control (a. AOPP; b. MDA; c. NO; d. catalase) (a). ****p<0.0001: AOPP tumor tissues vs. AOPP peritumoral stroma; (b) p>0.05: MDA tumor tissues vs. MDA peritumoral stroma; (c). ****p<0.0001: NO tumor tissues vs. NO peritumoral stroma; (d). *p<0,05: catalase tumor tissues vs. catalase peritumoral stroma.

On the other hand, the relationship between the smoking status and oxidative stress in tumor tissue was also analyzed and we noted a high level of AOPP (p<0.0001), NO (p<0.0001) and MDA (p>0.05) and low level of catalase (p<0.05) in the tumor tissue of smokers compared to control tissue (Table 3; Figure 2).

## Discussion

This study includes clinico-epidemiological analysis and redox imbalance evaluation in a small set of Algerian patients with LC.

In previous global and local studies, LC distribution has been widely studied and our findings are mostly in agreement with them. In the world, it was reported that the incidence of LC varies according to sex, geographical difference and histological type. It was also reported that LC is generally diagnosed at an advanced stage and occurs exponentially in late middle and older age and that NSCLC is the most common type, particularly with the adenocarcinoma sub-type [12]. In contrast, it was reported that in a province in northwest Algeria, the squamous cell carcinoma was the most common histological type [13]. Concerning causes of LC occurrence, it was indicated that active and passive smoking constitutes the main risk factor in the addition of professional exposure.

On the other hand, family history and predisposition of lung disease are clinically useful risk indicators [14]. In Algeria, it was previously indicated that LC is the most prevalent male cancer which affects subjects aged from 50 to 69 years old. LC is essentially caused by tobacco and other environmental factors and is diagnosed at an advanced stage (II and IV) with different symptoms such as chest pain, cough and so forth. [13,15].

Also, OS evaluation revealed an imbalance of redox status in LC patients which attests that the OS is implicated in carcinogenesis; those findings align with numerous studies [16-29].

Esme and al. and Malerba and al. showed that NO production rises in LC [16,17], by promoting DNA damage and delaying apoptosis [18]. To date, cigarette smoke is the main source of NO which is amply implicated in LC pathogenesis by inducing a chronic inflammation of the respiratory tract [19,20]. In addition, Kaynar and al. and Esme and al. indicated that MDA is a tumor promoter and co-carcinogen agent because of its high cytotoxicity and inhibitory action against protective enzymes [21,16]. In cancerous cells, lipid peroxidation products activate transcription factors and various intracellular signaling pathways [22]. The cigarette smoke could rise the pulmonary MDA level because smoking accelerates oxidative metabolism and then increases lipid peroxidation [23,24]. Furthermore, Pilgnatelli and al. reported a high level of oxidized proteins in smokers’ LC patients, due to increased oxidative species production during carcinogenesis [25]. MDA can combine with free amino groups of proteins to form adducts which alter protein properties [1]. Lipidic and proteic oxidative attacks promote carcinogenesis by aggravating DNA damage [26].

Moreover, some reports indicated that the antioxidant capacity of catalase is lower in lung tumor tissue compared to healthy tissues [19,22,27]. This diminution is due either to a rise of Reactive Oxygen Species production or the alteration of the antioxidant system [21]. To date, carcinogenesis is associated with a substantial reduction of antioxidants in smokers LC patients [28,29].

## Conclusions

LC is a common type of cancer which widely varies according to wide variations in demographic characteristics, histological type, and risk factors. The direct and indirect exposure (workplace, home and public places) to cigarette smoke constitutes the main LC risk factor. Regrettably, in Algeria there are no local laws which forbid smoking in public places; this present study could contribute to instituting laws that would reduce non-smokers’ exposure. To our knowledge, this is the first study that reports clinicopathological features and redox imbalance in Algerian lung cancer patients. Further prospective studies are needed to reaffirm our findings. This study will help to improve the outcome of lung cancer patients in Algeria.

## Conflict of Interest

The authors confirm that there are no conflicts of interest.
